# Ready-to-Eat Fish Cake Processing Methods and the Impacts on Quality and Flavor

**DOI:** 10.3390/foods11213321

**Published:** 2022-10-23

**Authors:** Caiyan Jiang, Yao Chen, Shuang Li, Shan Shang, Baoshang Fu, Lina Wang, Xiuping Dong, Pengfei Jiang

**Affiliations:** 1 School of Food Science and Technology, Dalian Polytechnic University, Dalian 116034, China; 2 National Engineering Research Center of Seafood, Dalian 116034, China; 3 Dalian Center for Certification and Food and Drug Control, Dalian 116034, China

**Keywords:** drying, ion migration chromatography, quality, scanning electron microscopy, sterilization temperature

## Abstract

This study aimed to compare tilapia fish cake drying and sterilization conditions (105, 115, and 121 °C) on the quality of the cakes. The impacts of volatile flavor substances, the chroma value, quality and structure characteristics, microscopic structure, and the types and content of volatile flavor substances were also analyzed. The results showed that after drying and sterilization, the L* value, W value and delta-E value of fish cakes decreased significantly from 77.12 to 64.77, 66.21 to 52.57, 10.46 to 24.50, respectively. However, a* value and b* value increased significantly from 0.30 to 6.97 and 24.85 to 30.89, respectively. The elasticity, hardness, and chewiness increased significantly with the drying process but decreased significantly with the increased sterilization temperature. Scanning electron microscopy results showed that the internal pores of the fish cakes became smaller, and the tissue structure was closer after drying. Gas chromatography—ion mobile spectrometry analysis identified a total of 36 volatile flavor compounds. Among these, ketones comprised the largest content, aldehydes represented the largest variety, and all volatile compounds contributed significantly to the flavor of fish cake. PCA results and nearest-neighbor fingerprint analysis showed that there were obvious differences in volatile flavor compounds between different treatments. In summary, this study conducted a detailed comparative analysis of the quality and flavor of fish cakes subjected to different processing methods. These findings contribute suggestions for sterilization temperatures in industrial production processes.

## 1. Introduction

Tilapia is the second most economic freshwater fish in the world [1] and is an important aquaculture species in China. Compared to other economic fish, tilapia has a high nutritional value due to the balance of protein and amino acids in its flesh [2]. Currently, tilapia products on the market are predominantly live fish and frozen fillets, which are less refined and processed products [3]. Dried fish fillets, canned fish, waxed products, and surimi products are the primary tilapia products requiring intensive processing. Despite all this, ready-to-eat tilapia products are limited. Therefore, enriching the range of tilapia products is a trending research topic and has great prospects for development.

As an essential part of food processing, sterilization is divided into two categories [4], including thermal sterilization (pasteurization, high temperature sterilization, and ultra-high temperature sterilization) and non-thermal sterilization (ultrasonic, ultraviolet radiation, ozone oxidation, and high hydrostatic pressure sterilization) [5]. Traditional thermal sterilization technology is widely used in industrial production due to its ease of operation, controllability, and low price. Chantakun et al. [6] demonstrated that different heat treatment methods, including pasteurization at a high temperature for a shorter time or at a low temperature for a longer time at 118 °C and 121 °C sterilization, had significant effects on the quality and characteristics of bird’s nest beverages. MA et al. [7] reported that different sterilization methods of Prunus mume juice have significant effects on the microbiological and physicochemical parameters, such as the degree of browning, color measurements, total phenolic content, reducing sugar, ascorbic acid, 5-hydroxymethylfurfural, amino acid nitrogen, total soluble solids (TSS), and pH value during storage.

Gas chromatography–ion mobility spectrometry (GC-IMS) is an emerging technique for the detection of flavor substances, which has the advantages of easy operation, high sensitivity, and fast detection [8,9,10]. It is used heavily in the food industry. Feng et al. [11] used GC-IMS to compare the differences between volatile flavor substances in yak milk powders under ultra-high-pressure sterilization and heat sterilization treatments. GC-IMS was used to analyze the characteristic volatile profiles of quinoas and the contents of volatile flavor substances were counted by normalization method [12]. There have been many studies that analyzed the optimal method of sterilization for surimi products, but there is considerably less research regarding the optimal sterilization temperature for tilapia thermal sterilization methods, which is used in industrial production at this stage. Therefore, the use of GC-IMS can effectively contrast the flavor changes of different treatments fish cakes.

This work investigates the effects of different treatments (untreated, dried, sterilized at 105 °C, sterilized at 115 °C, and sterilized at 121 °C) on the chromaticity values, textural properties, and microstructure of tilapia ready-to-eat fish cakes. Using GC-IMS, the differences of volatile flavor substances in fish cakes under different treatments were compared. Principal component analysis (PCA) was used to differentiate the fish cakes under different treatments. This study aims to provide a theoretical basis for the selection of sterilization conditions in the commercial production of tilapia fish.

## 2. Materials and Methods

### 2.1. Sample Preparation

Frozen tilapia was purchased from Hainan Xiangtai Fishery Co., Ltd. (Chengmai, China) After cutting into small pieces, the fish were washed with ice water and placed over crushed ice before processing. The fish were then put into a meat grinder and were ground two to three times. The fish cakes were made from a mixture of unrinsed surimi, 25% egg white powder, 30% mashed potatoes, 6% lard, and 100% water. The mixture was further ground for 2 min, placed into molds, and steamed at 90 °C for 25 min.

The samples were dried for 10 h at 10–15 °C in an air oven with an air speed of 1.5 m/s at 25% humidity. The dried samples were placed in a sterilizer with a reaction pressure of 0.12 MPa with experimental reaction temperatures of 105 °C, 115 °C, and 121 °C for 20 min, followed by cooling at room temperature. Fish cakes were set aside for further analysis.

### 2.2. Color Measurement

The color of fish cakes samples was measured according to Cao et al. [13]. The degrees of lightness (L*), redness (a*), and yellowness (b*) of fish cake samples were determined using a colorimeter (Ultra Scan PRO, HunterLab Inc., Reston, VA, USA). Fish cake samples were cut into thin 1 cm slices and placed vertically on a mirror port. Each sample measurement was repeated six times. Whiteness (W) and delta E (ΔE) were calculated according to the following formula:

W = 100 − ((100 − L*)^2^ + a*^2^ + b*^2^)^1/2^ (1)

ΔE = ((L − L_0_)^2^ + (a − a_0_)^2^ + (b − b_0_)^2^)^1/2^ (2)

### 2.3. Texture Profile Analysis

The texture profile analysis (TPA) of fish cake samples was determined according to methods from Mi et al. [14] The prepared fish cake samples were sliced to 10 mm, and a 22 mm diameter sample was taken with a meat picker and tested on a TA.XT.plus texture analyzer (Stable Micro Systems Co., Ltd., Godalming, Lammas Road, Vienna Court, UK) with a 50 mm cylindrical probe (P/50). The TPA was conducted using two 30% compression cycles with a 5 s interval between compressions. The pre-test, test, and post-test speed were 2, 1, and 1 mm/s, respectively, with a trigger force point of 5 g. Each sample analysis was repeated six times. Results are presented as averages.

### 2.4. Scanning Electron Microscopy

Scanning electron microscopy (SEM) of samples was obtained according to Chen et al. [15]. The filleted surimi samples were placed in a freeze dryer for lyophilization, removed, and placed under a thermal field emission scanning electron microscope for observation and imaging at a magnification of 200.

### 2.5. Gas Chromatography–Ion Mobility Spectrometry Analysis

The GC-IMS (FlavourSpec^®^, G.A.S. Instrument, Shandong Haineng Scientific Instrument Co., Ltd., Rizhao, China) conditions were slightly modified from Chi et al. [16].

Headspace injection conditions were as follows: chopped fish cake (2 g) was placed in a 20 mL headspace injection vial and incubated at 60 °C for 20 min prior to testing. Each sample was repeated three times. The injection needle temperature was 85 °C, and the incubation speed was 500 rpm.

The GC conditions were as follows: MXT-5 column (15 m × 0.53 mm, 1.0 μm) with a column temperature of 60 °C. The flow conditions of the carrier gas were varied as follows: 0–2 min 2 mL/min, 2–10 min 10 mL/min, 10–20 min 100 mL/min, 20–25 min 150 mL/min.

IMS conditions were as follows: IMS temperature was maintained at 45 °C, and both drift and carrier gases were nitrogen (purity ≥ 99.999%).

### 2.6. Statistical Analysis

The experimental values were analyzed by one-way analysis of variance (ANOVA) using SPSS 26.0 software (IBM, Armonk, NY, USA). The results are reported as the mean ± standard deviation, and significance was defined at *p* < 0.05. The volatile flavor compounds in the samples were analyzed using the built-in plug-ins of the GC-IMS instrument (Laboratory Analytical Viewer (LAV), Reporter, and Gallery Plot). LAV was used to view the analysis spectrum, with each point on the graph representing a volatile organic compound, and the GC × IMS Library Search used a two-dimensional cross qualitative method for qualitative analysis. The relative content of each substance was calculated by normalization method based on the ion peak volumes of the volatile flavor substances. The aroma descriptions for the volatile flavor components were compared from the literature.

## 3. Results and Discussion

### 3.1. Physical Properties

#### 3.1.1. Color

Four indicators (L*, a*, b*, W) were used to evaluate the color of food as the color parameters of international standard color and were used to further speculate the quality of food [17]. L* values indicate lightness, a* values represent red/blue, b* values indicate yellow/green, and W values stand for whiteness. The L* value of the dried fish cake was 76.19 ± 1.07, and the W value was 66.46 ± 1.18; both values were significantly lower than that of the untreated group (*p* < 0.05). However, the b* value (23.54 ± 0.79) was slightly higher than that of the untreated group (*p* < 0.05). Moreover, there was no significant difference in the a* value before and after drying. This might be due to the Maillard reaction between proteins and starches under dry conditions, resulting in a decrease in the lightness and whiteness values of the sample and an increase in the yellowness value [18].

**Figure 1:**
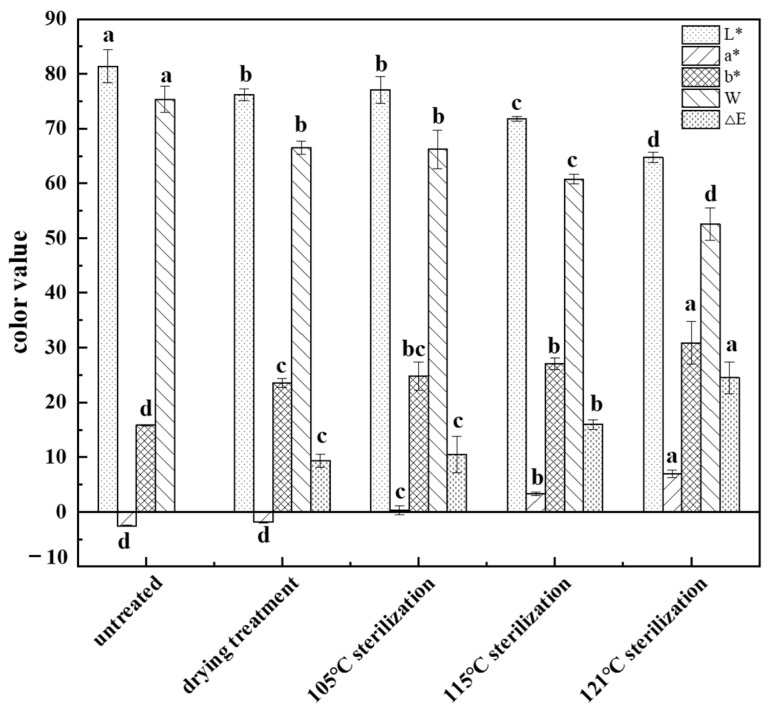
Influence of different treatments methods on the color of tilapia fish cake. The a, b, c and d represent significant differences among treatment groups, the same letter represents no significant difference between two treatment groups (*p* > 0.05), and different letters represent significant differences between two treatment groups (*p* < 0.05).

As shown in Figure 1, with the increase of sterilization temperature, the L* and W values gradually decreased, while the a*, b* and delta-E values gradually increased. The color difference of fish cakes gradually increased with the rise of sterilization temperature, indicating that the surface color of fish cakes kept deviating from the initial point, and there were significant differences among the delta-E values of fish cakes treated at different sterilization temperatures, with the delta-E value of 105 °C sterilization being the closest to that of dried fish cakes, and there was no significant difference between them(*p* > 0.05). This was probably due to the contraction and disintegration of myofibrils during the heat treatment, which led to Maillard reactions between sugars and proteins and Browning reactions between proteins and fats [19]. With the sterilization temperature increased to 121 °C, the L* (64.77 ± 0.97) and W (52.57 ± 2.97) values of the fish cake reached the lowest value, while the a* (6.97 ± 0.72) and b* (30.89 ± 3.87) values reached the highest level. Additionally, there was a significant difference (*p* < 0.05) with other treatment groups.

The deeper color in fish cakes might be caused by a more intense Maillard reaction associated with increasing temperature. Zhu et al. [20] found that the L*, a*, and b* values of pufferfish fillets were dramatically affected by different drying methods, and the color values of fish fillets under high temperature drying conditions were significantly higher than those under low temperature conditions, which mainly resulted from a Maillard reaction. In summary, 105 °C was chosen as the optimum sterilization temperature, which could play a sterilization effect while maintaining the original color of fish cake, being more attractive to consumers.

#### 3.1.2. Texture Analysis

Texture is an important indicator of seafood quality and consumer acceptance. The textures of the fish cake under different treatments are listed in Table 1. The hardness (2124.39 ± 255.03), chewiness (1625.14 ± 203.73), and cohesiveness (0.83) of the dried fish cakes were significantly higher than those of the untreated fish cakes (*p* < 0.05), but there were no significant differences in the springiness of the dried fish cake (*p* > 0.05). This might be due to the loss of moisture in the fish cake during the drying process, resulting in a tighter internal structure, which becomes harder and chewier. Becker et al. [21] also found that the hardness and chewiness of fish cakes were higher at a drying temperature of 55 °C, which was related to the degeneration of protein and contraction of myofibrils.

With increasing sterilization temperatures, there were no obvious changes in the springiness, cohesiveness presented a gradual upward trend, but the hardness and chewiness showed an increasing trend followed by a decreasing trend. The hardness and chewiness under sterilization at 105 °C were 4147.75 ± 444.90 and 3284.86 ± 412.35, respectively, which were dramatically different from other groups (*p* < 0.05). There was no significant difference between hardness and chewiness under 115 °C and 121 °C sterilization conditions (*p* > 0.05). Zhang et al. [22] also found that the hardness, springiness, cohesiveness, and chewiness of surimi gel decreased when the temperature increased from 100 °C to 121 °C.

#### 3.1.3. SEM Analysis

**Figure 2:**
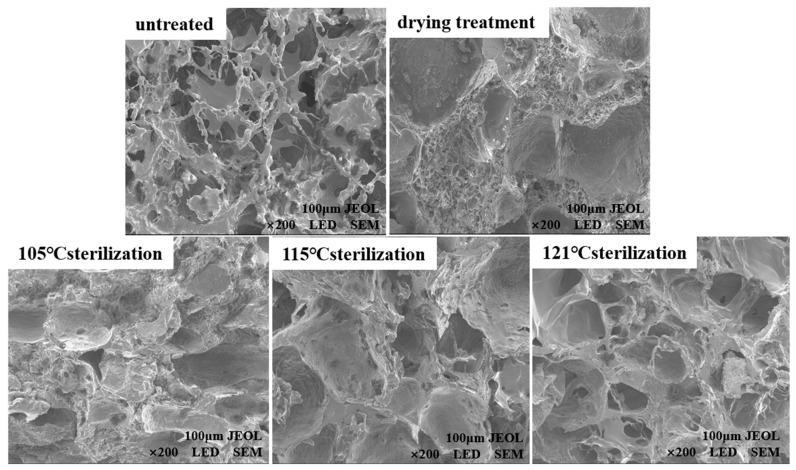
Comparison of microstructure of tilapia fish cake under different treatments methods.

SEM micrographs of fish cakes of different treatments are displayed in Figure 2. The surface of the untreated fish cake was uneven and rough, and there were many irregular pores inside. After hot air drying and thermal sterilization treatment, the holes in the fish cake were reduced with a more compact structure. Wang et al. [23] found that water molecules were more easily stored in small pores, thus in the gel system, the three-dimensional network structure, which has small and uniform holes, can effectively lock water molecules. The fish cake exhibited good gel properties. This could be attributed to the protein molecules within the fish cakes that are denatured under high temperature conditions, and the interaction force between the molecules is changed to form aggregates [24]. As the sterilization temperature increased from 105 °C to 121 °C, the pores inside the fish cake gradually increased, which might be due to the further destruction of the higher-order structure of the proteins causing the aggregation of the proteins to become more serious [25].

### 3.2. GC-IMS Analysis

#### 3.2.1. GC-IMS Spectrum Analysis of Flavor Components of Fish Cakes under Different Conditions

**Figure 3:**
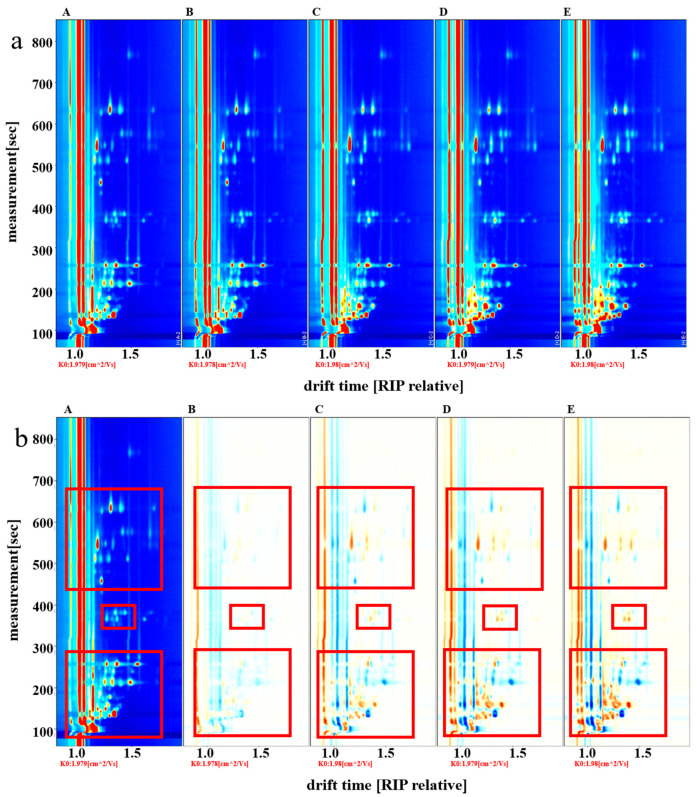
Two-dimensional GC-IMS spectra of volatile organic compounds in tilapia fish cakes with different processing methods. (**a**) original spectrum; (**b**) differential contrast spectrum. (A) untreated fish cake; (B) drying treatment fish cake; (C) 105 °C sterilization fish cake; (D) 115 °C sterilization fish cake; (E) 121 °C sterilization fish cake. Red boxes were the difference of volatile organic compounds among different processing methods.

The GC-IMS two-dimensional spectra of volatile flavor compounds in fish cakes under different treatments are shown in Figure 3a. There were significant differences in the flavor compounds of the fish cakes under different treatment conditions, and significant amounts of ammonia gas were detected in all fish cake samples, while a large amount of trimethylamine was detected in sterilized fish cakes. With increasing sterilization temperatures, the content of trimethylamine showed an increasing trend, which could be explained by a large amount of trimethylamine oxide contained in the fresh fish. Under high-temperature conditions, trimethylamine oxide was rapidly decomposed to form trimethylamine [26]. To compare the differences in a clearer fashion, the spectrum of the untreated fish cake sample was selected as the reference, and the same parts of other treatment groups were subtracted from this basis (Figure 3b). If they contain the same volatile organic compounds, the background after deduction is white; red indicates that the content of the substance is higher than the reference, and blue means that the content of the substance is lower than the reference. The content of flavor substances in fish cakes did not change much after the drying treatment. Nevertheless, under different sterilization temperatures, the contents of flavor substances were significantly different, and an increasing or decreasing trend was observed in the contents of different volatile flavor substances. The highest volatile flavor substance content was identified in the samples sterilized at 121 °C. This might be the result of the thermal treatment process promoting the release of some flavor substances, which increases the content of flavor substances in the fish cakes [27]. These findings are similar to Bai et al. [28], which indicated that the content and types of flavor substances in cooked Antarctic krill and white shrimp were significantly increased. Nonanal, benzaldehyde, and limonene were produced, and pyrazines, aldehydes, ketones, and alcohols increased in steamed and cooked shrimp meat. Furthermore, the free glycine level increased from 86.48 to 687.12 mg/100 g in shrimp after steaming.

#### 3.2.2. Qualitative Analysis of Flavor Components by GC-IMS of Fish Cakes Subjected to Different Processing Conditions

**Figure 4:**
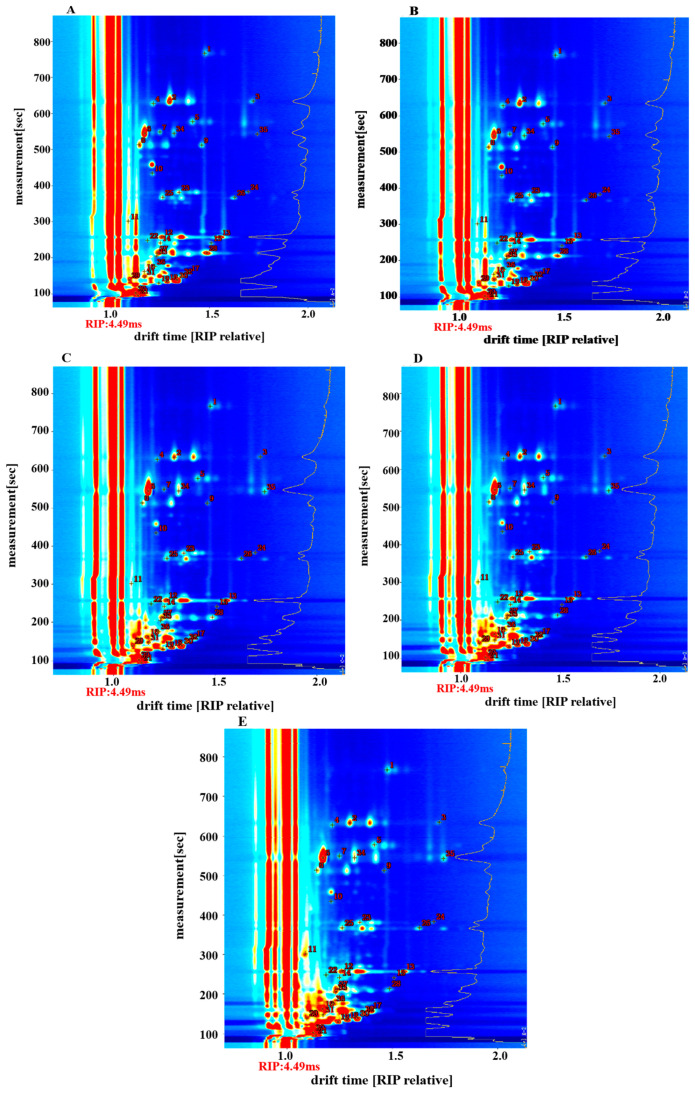
Qualitative analysis by GC-IMS of volatile flavor compounds in tilapia fish cakes with different treatments methods. (**A**) untreated fish cake; (**B**) drying treatment fish cake; (**C**) 105 °C sterilization fish cake; (**D**) 115 °C sterilization fish cake; (**E**) 121 °C sterilization fish cake. The lines represented the drift time of volatile components. Each number represented a compound.

Qualitative analysis of flavor substances in fish cakes was conducted by comparisons with the built-in database of the flavor instrument, according to the retention time and migration time of the flavor substances [29]. Forty-four signal peaks were detected, and thirty-six flavor substances, including monomers and dimers, were identified in the samples. These substances comprised ketones, which were divided into seven categories, namely alcohols, esters, aldehydes, olefins, pyrazine, and furfuran. Monomers and dimers had the same chemical formula in the same substance and differed only in form. The figures in Figure 4 correspond to the compounds in Table 2.

#### 3.2.3. Fingerprint Analysis by GC-IMS of Volatile Components of Fish Cakes under Different Conditions

**Figure 5:**
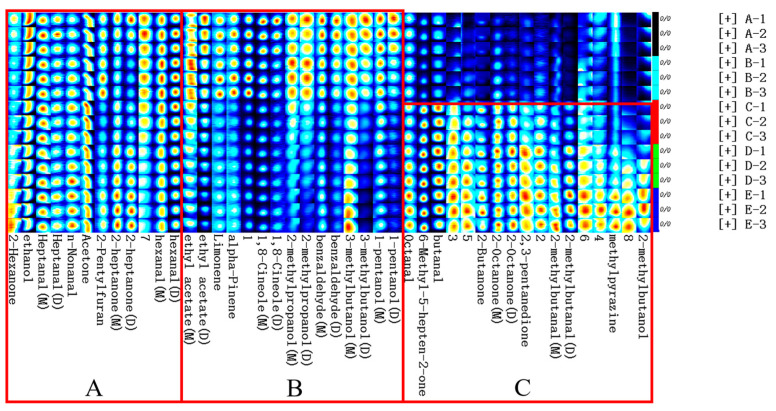
Fingerprint of volatile organic compounds in tilapia fish cakes with different treatment methods. Numbers represent unidentified components. M and D in the parentheses represent the monomer and dimer of the substance, respectively.

To further explore the differences between volatile flavor compounds in fish cakes under different processing treatments, the fingerprint analysis of flavor compounds in fish cakes was performed using the Gallery Plot plug-in of the LAV software. As shown in Figure 5, all signals contained in each sample were displayed in the same row, and the signals of one flavor in different samples were shown in the same column. Meanwhile, higher levels of the substance are indicated when more red color is present. Each sample was measured three times in parallel.

As shown in Figure 5, the common volatile flavor substances in fish cakes under five different conditions are displayed in area A, including 2-hexanone, ethanol, heptanal (monomer, dimer), n-nonanal, acetone, 2-pentylfuran, 2-heptanone (monomer, dimer), and hexanal (monomer, dimer). The flavor substances in untreated and dried treatment fish cakes were mainly concentrated in region B, which was lesser or not detected in sterilized fish cakes, containing ethyl acetate (monomer, dimer), limonene, alpha-pinene, 1,8-cineole (monomer, dimer), 2-methylpropanol (monomer, dimer), benzaldehyde (monomer, dimer), 3-methylbutanol (monomer, dimer), and 1-pentanol (monomer, dimer). The flavor substances in fish cakes after sterilization were mainly focused on region C, including octanal, 6-methyl-5-hepten-2-one, butanal, 2-butanone, 2-octanone (monomer, dimer), 2,3-pentanedione, 2-methylbutanal (monomer, dimer), methylpyrazine, and 2-methylbutanol.

**Figure 6:**
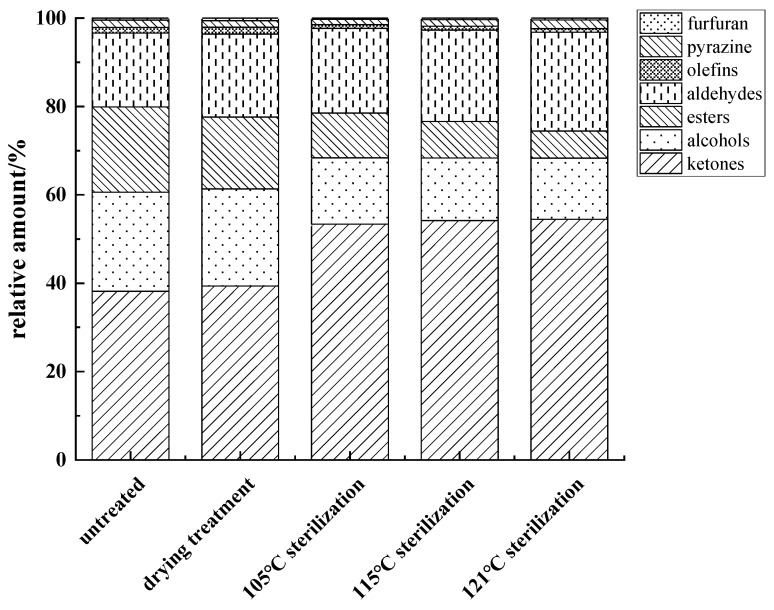
Relative content changes of volatile components in tilapia fish cakes with different processing methods.

The content of various flavor substances in fish cakes was analyzed and compared by the peak volume normalization method. The highest flavor substance content in fish cakes was ketones (36.21–51.30%), which was caused by the degradation of amino acids and the Maillard reaction, in which the contents of acetone and 6-methyl-5-hepten-2-one were higher (Figure 6). There was no significant difference in acetone (spicy) content among different treatments, but the content of 6-methyl-5-hepten-2-one (fruity and fragrant) in the sterilized fish cake increased significantly, which contributed positively to the overall flavor of the fish cake. However, the ketones had a high threshold and, therefore, contribute less to the overall flavor of the fish cake.

The alcohol content of the untreated fish cakes was the highest and decreased by 8.23% after sterilization treatment, which might be related to the reaction between alcohol and acid. The content of ethanol in fish cakes was relatively high, which is reduced to an extent after sterilization. The content of 1,8-cineole (camphoraceous and herbal) was followed only by ethanol and exemplified a decreasing trend after sterilization.

Esters, generated by the esterification of carboxylic acid and alcohol, are ethyl acetate monomers and dimers (fruit aroma) [30], and the content in fish cakes was 5.79–18.27%. The threshold value of the ester was lower, but the contribution to flavor substances was greater, which could give a unique ester flavor to fish cakes. With increasing sterilization temperatures, the ester content decreased, indicating that higher sterilization temperature would lead to the loss of flavor substances in food.

Aldehydes were mainly formed by the oxidation and decomposition of fats, and the content in fish cakes ranged from 15.88–21.05%; the threshold of aldehydes was low, which made a great contribution to the overall fish cake flavor. A high content of hexanal (grassy, fatty), benzaldehyde (almondy, fruity), 2-methylbutyraldehyde, and butyraldehyde (spicy) were detected as aldehydes. The progressive increase in the content of 2-methylbutyraldehyde and butyraldehyde adversely affected the flavor of fish cakes, which was accompanied by increased sterilization temperatures.

Olefins (0.77–1.42%) comprised limonene and α-pinene, and pyrazines (1.12–1.87%) was composed of methylpyrazine. The only furan flavor substance (0.28–0.53%) was 2-pentylfuran which provided bean and fruit flavors. Because the relative contents of olefin, pyrazine, and furan in the fish cake were small and the different treatments had little effect on their contents, the overall flavor of the fish cake was generally not affected. As previously mentioned, higher sterilization temperatures not only lead to the loss of positive flavor substances, but they also produce negative flavor substances, affecting the final flavor of fish cakes. Xie et al. [31] studied the effects of different heat treatment temperatures on the flavor compounds of boiled, salted duck and found that low temperature heat treatments better controlled the changes of flavor compounds, similar to the findings of this study.

### 3.3. PCA and Nearest-Neighbor Distances Analysis

**Figure 7:**
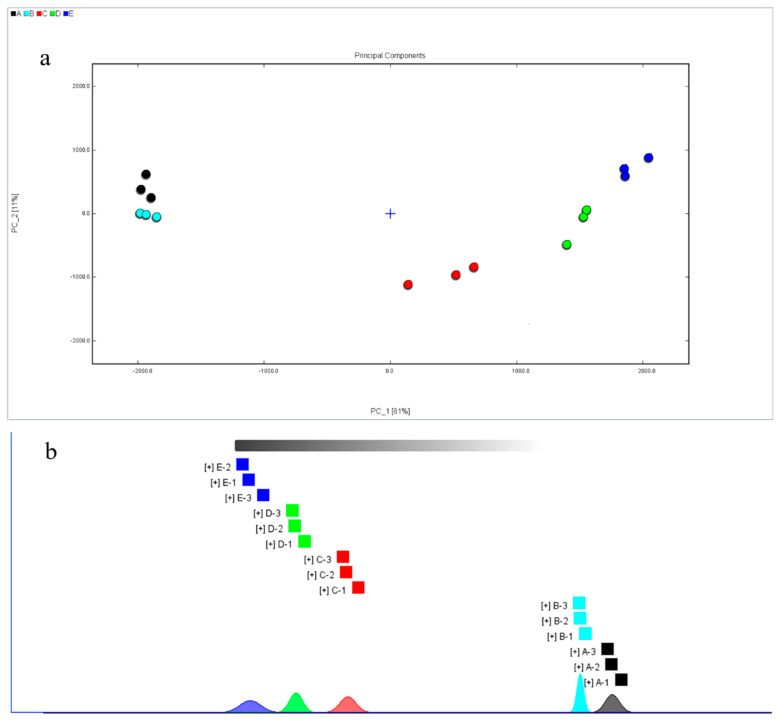
PCA score (**a**) and nearest neighbor distances (**b**) of volatile organic components in tilapia fish cakes with different treatment methods.

PCA is a dimensionality reduction method for analyzing large amounts of data. Currently, this method in employed in various fields of material identification and classification. For example, it is used in the identification of volatile substances in Chinese dry-cured hams from different regions, assessing peaches with different preservation methods, and analyzing yak milk powder processed by different drying methods [32,33,34]. In this study, PCA analysis was performed on 36 volatile flavor compounds in fish cakes under different processing conditions. The contribution rate of PC1 was 81%; the contribution rate of PC2 was 11%, and the cumulative contribution rate reached 92%, which indicated that the detected flavor substances are highly representative of the information in the fish cake (Figure 7a). The flavor substances in fish cakes with the same treatment conditions were roughly concentrated in one classification area. The flavor substances in the untreated and dried fish cakes were close, indicating little difference in flavor substances between them. Additionally, after the samples were sterilized, the flavor substances were different. Moreover, the flavor substances of fish cakes treated with different sterilization temperatures were significantly different, indicating that the flavor substances of fish cakes treated with sterilization change significantly. Overall, there was no clear overlap between the flavor substances in the fish cakes under different processing treatments, which indicates that the use of PCA could better distinguish the fish cakes under different treatments.

The nearest-neighbor analysis showed the same results as did PCA (Figure 7b). The volatile organic compounds of the fish cakes before and after drying did not change significantly, but the volatile organic compounds changed greatly after high temperature sterilization. Concurrently, there were differences in the volatile organic compounds in the fish cakes sterilized at three different temperatures. Thereby, the lower sterilization temperature can maintain the original flavor of the fish cake as much as possible.

## 4. Conclusions

Sterilization is an essential step in food processing. This study showed that different processing methods have different effects on the quality and flavor of tilapia instant fish cakes.

In terms of physical characteristics, color, texture index, and the microstructure of the fish cakes after sterilization changed significantly. With increasing sterilization temperatures, the L* and W values decreased significantly and the a* and b* values increased significantly. The hardness and chewiness of fish cakes showed a decreasing trend with the increase of sterilization temperature. The increase of the sterilization temperature caused the pore size between the tissues to increase gradually. In terms of flavor, the flavor substances in fish cakes after sterilization were mainly octanal, 6-methyl-5-hepten-2-one, butanal, 2-butanone, 2-octanone (monomer, dimer), 2,3-pentanedione, 2-methylbutanal (monomer, dimer), methylpyrazine, and 2-methylbutanol. Through the analyses of the color, texture, microstructure, and various flavor substance contents in fish cakes, the results showed that the higher sterilization temperatures changed the original color and the quality of fish cakes, which caused the loss of positive flavor substances. Additionally, more negative flavor substances were produced, and so 105 °C was chosen as the optimal sterilization temperature. In summary, under three different sterilization temperatures, 105 °C sterilization can prepare better quality tilapia instant fish cakes, which provides data references for industrial production of tilapia products.

## Figures and Tables

**Table 1:** Changes in the texture of tilapia fish cakes under different treatments.

Condition	Hardness	Springiness	Cohesiveness	Chewiness
untreated	1008.67 ± 55.66 ^d^	0.95 ± 0.03 ^a^	0.81 ^d^	778.61 ± 55.01 ^d^
drying treatment	2124.39 ± 255.03 ^c^	0.92 ± 0.01 ^a^	0.83 ^c^	1625.14 ± 203.73 ^c^
105 °C sterilization	4147.75 ± 444.90 ^a^	0.94 ± 0.04 ^a^	0.84 ^b^	3284.86 ± 412.35 ^a^
115 °C sterilization	3451.19 ^b^	0.94 ^a^	0.86 ^a^	2817.08 ^b^
121 °C sterilization	3252.04 ± 203.04 ^b^	0.93 ± 0.01 ^a^	0.86 ^a^	2614.42 ± 171.73 ^b^

Data are expressed as the mean ± SD (*n* = 6). Values within a row with different superscripts are significantly different (*p* < 0.05).

**Table 2:** Volatile compounds detected from tilapia fish cakes via GC–IMS.

No.	Compounds	CAS#	Formula	MW	RI	RT [s]	DT [ms]	Thresholds (mg/100 mL)	Comment
1	n-Nonanal	C124196	C_9_H_18_O	142.2	1103.3	766.102	1.47903	0.01	
2	1,8-Cineole	C470826	C_10_H_18_O	154.3	1041.7	632.512	1.30201	-	Monomer
3	1,8-Cineole	C470826	C_10_H_18_O	154.3	1042.4	633.818	1.72512	-	Dimer
4	Limonene	C138863	C_10_H_16_	136.2	1038.8	626.856	1.21739	0.1	
5	Octanal	C124130	C_8_H_16_O	128.2	1012.4	577.25	1.41819	0	
6	6-Methyl-5-hepten-2-one	C110930	C_8_H_14_O	126.2	994.0	544.18	1.17436	0.68	
7	2-Pentylfuran	C3777693	C_9_H_14_O	138.2	995.9	548.096	1.25181	0.06	
8	benzaldehyde	C100527	C_7_H_6_O	106.1	978.4	512.415	1.14425	3.5	Monomer
9	benzaldehyde	C100527	C_7_H_6_O	106.1	977.9	511.545	1.46408	3.5	Dimer
10	alpha-Pinene	C80568	C_10_H_16_	136.2	934.9	433.628	1.21337	0.14	
11	methylpyrazine	C109080	C_5_H_6_N_2_	94.1	835.9	300.659	1.09188	600	
12	hexanal	C66251	C_6_H_12_O	100.2	793.2	257.954	1.26268	0.04	Monomer
13	hexanal	C66251	C_6_H_12_O	100.2	791.9	256.759	1.55567	0.04	Dimer
14	1-pentanol	C71410	C_5_H_12_O	88.1	774.8	240.932	1.25319	40	Monomer
15	1-pentanol	C71410	C_5_H_12_O	88.1	774.5	240.633	1.51178	40	Dimer
16	2-methylbutanal	C96173	C_5_H_10_O	86.1	665.9	161.822	1.17209	0.01	Monomer
17	2-methylbutanal	C96173	C_5_H_10_O	86.1	658.7	158.453	1.3988	0.01	Dimer
18	butanal	C123728	C_4_H_8_O	72.1	601.4	133.797	1.28837	0.02	
19	2-Butanone	C78933	C_4_H_8_O	72.1	589.1	129.049	1.24595	354	
20	Acetone	C67641	C_3_H_6_O	58.1	509.1	101.936	1.12887	8.32	
21	ethanol	C64175	C_2_H_6_O	46.1	483.5	94.547	1.14128	9500	
22	2-Hexanone	C591786	C_6_H_12_O	100.2	782.2	247.897	1.18847	5.6	
23	Heptanal	C111717	C_7_H_14_O	114.2	901.1	380.762	1.34963	0.03	Monomer
24	Heptanal	C111717	C_7_H_14_O	114.2	902.7	383.137	1.69934	0.03	Dimer
25	2-heptanone	C110430	C_7_H_14_O	114.2	891.8	367.459	1.26607	30	Monomer
26	2-heptanone	C110430	C_7_H_14_O	114.2	891.8	367.459	1.63126	30	Dimer
27	3-methylbutanol	C123513	C_5_H_12_O	88.1	741.5	211.98	1.23925	0.04	Monomer
28	3-methylbutanol	C123513	C_5_H_12_O	88.1	742.7	212.985	1.4887	0.04	Dimer
29	ethyl acetate	C141786	C_4_H_8_O_2_	88.1	611.8	137.98	1.09743	0.05	Monomer
30	ethyl acetate	C141786	C_4_H_8_O_2_	88.1	613.8	138.784	1.3402	0.05	Dimer
31	2-methylpropanol	C78831	C_4_H_10_O	74.1	634.7	147.632	1.17418	65.05	Monomer
32	2-methylpropanol	C78831	C_4_H_10_O	74.1	638.4	149.242	1.36382	65.05	Dimer
33	2-methylbutanol	C137326	C_5_H_12_O	88.1	732.2	204.545	1.23515	0.159	
34	2-Octanone	C111137	C_8_H_16_O	128.2	994.2	544.639	1.32285	0.5	Monomer
35	2-Octanone	C111137	C_8_H_16_O	128.2	993.1	542.281	1.7448	0.5	Dimer
36	2,3-pentanedione	C600146	C_5_H_8_O_2_	100.1	692.2	175.419	1.22603	-	

MW, Molecular weight of identified volatile compounds; RI, Retention index in the MXT-5 column; RT, Retention time of identified volatile compounds; DT, Drift time in the drift tube.

## Data Availability

Data is contained within the article.
